# Acute stress disorder and the associated factors among traumatized patients admitted at Felege-Hiwot and the University of Gondar comprehensive specialized hospitals in Northwest Ethiopia

**DOI:** 10.1186/s12888-022-03961-9

**Published:** 2022-05-02

**Authors:** Asnakew Worku, Getachew Tesfaw, Berhanie Getnet

**Affiliations:** 1 University of Gondar, Gondar, Ethiopia; 2 Departments of Psychiatry, College of Medicine and Health Science, University of Gondar, P. O. Box: 196, Gondar, Ethiopia

**Keywords:** Acute stress disorder, Traumatized patients, Ethiopia

## Abstract

**Background:**

Acute stress disorder is the main factor of impairment in multiple areas of functioning that affects almost all age groups and which also influences mental and physical health. However, it negatively impacts the quality of life and social activities. The empirical evidence about probable acute stress disorder (ASD) and its associated factors is not available in Ethiopia to date. Therefore, the present study was aimed at identifying the magnitude and associated factors of probable ASD among traumatized patients in order to plan and render informed intervention for these vulnerable people.

**Methods:**

An institutional-based cross-sectional study was conducted at Felege-Hiwot and the University of Gondar comprehensive specialized hospitals from March 11/2020 to April 20/2020, by using a structured and semi-structured questionnaire. Systematic random sampling was used to recruit a total of 422 patients. The standard acute stress disorder scale was used to identify the prevalence of acute stress disorder by employing a face-to-face interview. Bivariate and multivariate logistic regression analysis was used to identify associated factors with probable acute stress disorder. Statistical significance was declared on 95% of confidence intervals (CI) at P < 0.05.

**Results:**

The prevalence of probable acute stress disorder was found to be 45% (95% CI: 40.2 to 49.6). In the multivariate logistic analysis; exposure to past history of trauma (AOR = 3.46, 95%, CI: 1.01–11.80), past psychiatry illness (AOR = 3.02, 95% CI: 1.15–7.92), anxiety (AOR = 2.38, 95% CI: 1.30–4.38), poor social support (AOR = 4.07, 95% CI: 2.20–7.52) and moderate (AOR = 4.56, 95% CI:2.44–8.52), and sever perceived threat to life (AOR = 2.75, 95% CI: 1.64, 4.60) were factors significantly associated with probable acute stress disorder.

**Conclusion:**

Findings of this study indicated that the prevalence of probable acute stress disorder among study participants exposed to multiple forms of traumatic events was considerably high. History of trauma and past psychiatric illness, poor and moderate social support, and moderate perceived stress were factors significantly associated with probable acute stress disorder. The ministry of health and other concerned health organizations may find the current finding useful for early detection, prevention, and intervention strategies to minimize the factor of acute stress disorder in trauma survivors.

## Introduction

Trauma has negative impacts which include morbidity and mortality of patients with differences in the impact of severity across ages and severity of an injury [1]. It is also a psychological shock to individuals who are exposed to an event that involves actual and unexpected death or serious injuries immediately after individuals have experienced an event [2].

According to the World Health Organization (WHO) estimated, road traffic-correlated death would increase 2000–2020 with 80% in low-and middle-income nations [3]. The evidence showed that traumatic experiences related to accident accounted for 32% in sub-Saharan Africa in the year 2015 [4]. The evidence from East Africa, specifically in Sudan in 2014, and Tanzania in 2005 showed that there is a significant developing burden of traumatic events [5, 6]. Being one of the developing nations, an accident is a typical general health problem in Ethiopia [7].

Acute stress disorder may happen after patients have encountered dangerous circumstances as a consequence of exposure to traumatic events, which they began to respond with unexpected dread or weakness to it [8]. It is characterized by the accompanying symptoms like intrusion, dissociation, avoidance, and expanded excitement in which these manifestations happen within two days to a month, and hence individuals with ASD are experiencing distress on significant areas of their everyday life. It is a risk factor for the development of post-traumatic stress disorder (PTSD), which is related to disability in personal satisfaction, social working, and large well-being [9–11]. The study showed that an unexpected traumatic situation could not only contribute physical injury but also might lead to survivors an increased risk for psychiatric co-morbidity especially acute stress disorder in Australia as reported in 1999 [12]. The study investigated with road traffic accidents indicate that those males and females who met acute stress disorder within one month could be diagnosed with PTSD after six months accounted for 57% and 92%, respectively [13]. In the same study, it was also evidenced that 83% of victims of hostility who are diagnosed with acute stress disorder within one month following crime still experienced from PTSD six months later. As per the 2013 DSM-5 diagnostic criteria, the prevalence of ASD in people who were exposed to serious traumatic stress depends on the severity and persistence of the trauma and the degree of exposure to it [14]. One study conducted in United States indicates that physical injury was associated with the psychiatric complications, which are risk for personal health problem; estimated that 12 to 16 percent of survivors with history of traumatic injury have developed acute stress disorder in 2005 [15]. The epidemiological study that investigated the prevalence and risk factors of acute stress disorder among trauma exposure patients demonstrated that between 5 and 20% prevalence of ASD with traumatic events varied across studies because of heterogeneity of population, a time when a study conducted and method of assessment used; and the evidence that the risk factors of ASD are the history of psychiatric disorder, history of traumatic exposures before recent exposure, female gender, trauma severity, and the avoidant coping style in 2019 [16]. A longitudinal study report based on DSM-IV criteria reported that 10% of the victim from among 129 patients exposed for traumatic injuries were suffering from ASD in Australia [17]. Some literatures indicate that acute stress disorder, and anxiety are the most widely recognized as mental problems after encountering trauma incidences(tragedies), which shatters the psychological well-being of an individual that is impacting his/her physical, psychological, social, and occupational functioning shortly after the accident [18, 19].

While there are numerous research outputs on risks and protective factors of people exposed to PTSD, there is a paucity of research evidence regarding those people who have developed acute stress disorder, including its associated risk and protective factors. One review study reported that people who were exposed to an injury, people with earlier PTSD as well as other form of mental problems are at risk for developing ASD when faced with another unpleasant stress [20]. Another study reported that motor vehicle accident survivors, depression, history of mental treatment, history of PTSD, and earlier traumatic events could be the risk factors of ASD [21]. Furthermore, other factors that may lead for the development of ASD include subjective distress during painful circumstances, and being a female by gender [22].

To the best of our knowledge, there is no other study to date, which was carried out acute stress disorder and its associated factors in traumatized patients admitted for care in hospitals. Hence, acute stress disorder was not sufficiently studied in traumatized patients who are exposed to serious situations like physical assaults or injury, car accident, burn, shooting with a gun, and violent events encountered to admitted patients. Acute stress disorder is the main factor of impairment in aspects of multiple functioning in those traumatized people which results in a serious negative and counterproductive impact on patients’ social relationships, their quality of life, and other adverse consequences on their health in general [17]. In Ethiopia, acute stress disorder has not been studied among traumatized patients who have undergone through serious situations like physical injury, car accident, burn, shooting with a gun and violent events neither among inpatients admitted and receiving ongoing care in hospitals nor outpatient settings. Besides, ASD was not studied at other institutions providing healthcare service centers in Ethiopian cultural contexts, though there are lots of traumatic incidences in every corner of the country. Still, people who are exposed for a variety of life-shattering traumatic incidences (tragedies) were investigated for the status of their mental well-being with respect different psychiatric disorders like PTSD, anxiety, and substance abuse, very little attention has been paid to identify acute stress disorder and its associated factors in people exposed to a different traumatic situation, which is common problem in African and Ethiopian context in particular. Therefore, the current study was conducted to assess the magnitude and associated factors of probable acute stress disorder among physically traumatized patients at the University of Gondar and Felege-Hiwot comprehensive specialized hospital in the Northwest, Ethiopia.

## Methods and materials

### Study design

Institutional-based cross-sectional study design was employed among traumatized patients who were exposed for multiple forms of physical trauma and admitted in patient care unit in different wards from March 2020 to April 2020. The research was conducted at Felege-Hiwot and Gondar University Comprehensive Specialized referral hospitals situated at Bahir Dar and Gondar, respectively.

### Study context

The study was conducted in two big referral hospitals (i.e. University of Gondar Specialized Hospital and Felege-Hiwot Comprehensive Specialized Hospital), having a wider catchment for a large proportion of people coming from different health institutions through referral in northwest Ethiopia. The study sites were selected based on criteria of hosting Orthopedic and trauma wards providing health care treatment for victims of physical trauma and these two hospitals are the largest hospitals providing service for the target population of the current study.

The city of Gondar where part of the current study was conducted is known for attracting the largest number of tourists, is situated in Northwestern Ethiopia at a distance of 724 km from Addis Ababa (the capital) and 175 km from the nearby city of Bahir Dar. The surrounding society in Gondar and Bahir Dar, in particular, are recently exposed for multifactorial problems of human rights violations, such as domestic abuse, aggression, looting and consequently prone to physical and psychological trauma. And the only comprehensive specialized hospitals which delivered different medical services to ensure that patients are seeing the correct providers for the correct problems in Amhara region which increases the volume of patients in the two setting to get appropriate sample size for this study. The University of Gondar Comprehensive Specialized Hospital is among the largest tertiary teaching hospital in Ethiopia. It was established in 1954, and hence this is the oldest medical training institution in the country. This comprehensive specialized hospital is providing health care for an estimate of around 5 million people in the catchment areas and it has been rendering health care services for more than six decades. Still this hospital has continued to provide a wider medical and health care service to wider catchment areas of northern western Ethiopia.

The previous study showed that the hospital comprises of a total of 579 health professionals, from whom 262 were nurses, 123 were physicians and the remaining belong to other health professionals. The previous study showed that 579 health professionals, from whom 262 nurses, 123 were physicians, and the remaining belongs to other professionals [23]. Specifically, in this study setting there were 67 nurses and 28 physicians who have been providing health care service for 815 patients having physical trauma per month, whereas 9,780 patients were admitted in inpatient wards for annum (Orthopedic ward, and Trauma ward).

Felege-Hiwot comprehensive specialized hospital’s found in the capital city of the Amhara Regional state Bahir Dar which is located at a distance of 553 km far from, Addis Ababa the capital city of Ethiopia. Felege-Hiwot was established in 1963 as a district hospital and upgraded into a referral hospital in 2002 and again to promote into a comprehensive specialized hospital in 2019. This grand hospital gives clinical services for 5.5 million people in the catchment area.

More specifically, in this study setting (surgical and orthopedics ward), there were 27 nurses and 12 physicians who have been providing treatment and care for 400 adult trauma patients per month and for 4,800 adult trauma patients per annum. Patients who exposed to physical traumatic events (i.e. those who experienced a trauma within 2–30 days) and those patients aged 18 years old and above were included. Those who have a severe illness such as disorientation or those who was in a state of coma were excluded.

### Sample size calculation and sampling technique

The sample size was calculated by using a single population proportion formula computed using Epi-info version seven with a 95% CI, a 5% margin of error, and considering the prevalence for ASD of 50% due to lack of published work in Ethiopia. Having assumed a 10% of non-response rate, 422 participants were recruited randomly by using systematic random sampling technique. The study participants were selected from the patients’ charts/ i.e. registration during admission) registration books by using a systematic random sampling technique from the two hospitals. Participants were proportionally selected from the independent hospitals, whose allotment was determined proportionally based on their admission to the respective wards per-month. Based on the information obtained during the study period from the inpatients units from the two hospitals, there were 1,215 traumatized patients admitted monthly in these wards. In order to fill the required sample size of 422 individuals, the total sample size selected from Felege-Hiwot hospital was 139 and the sample size selected from Gondar University specialized hospital was 283. In order to select participants from the two study sites, the sampling interval was allocated proportionally to each study setting, which was determined by dividing the total study population during one month of data collection period. The first study subject was selected by lottery method. Then participants were selected based on every two intervals starting from the first randomly selected participant until the required number of the participants in each hospital was attained.

### Study variables

#### Data source and measurements

Data were collected using an interviewer-administered questionnaire, which contains several other explanatory variables-including socio-demographic characteristics (age, sex, educational background, marital status, occupation, and others), clinical factors (anxiety, medication, history of psychiatry illnesses, chronic medical illnesses, type of trauma, history of trauma), psychosocial factors (social support and perceived life-threatening), and substance use factors (alcohol, tobacco, khat, Cannabis). Data for all these variables were collected using structured and semi-structured questionnaires, the following instruments were employed: Anxiety symptoms were collected using a standard questionnaire (GAD-7 scale) which has a sensitivity of 89% and specificity of 82% [24]; this tool was validated in Ethiopia and its Cronbach’s alpha value found to be 0.82 (*n* = 219) [25]. In the current study, internal consistency of alpha value was found to be 0.71 and it measures patient-reported GAD-7 based on DSM-IV criteria with seven items on a four-point Likert scale(0 = not at all to 3 = nearly every day) and a two week frame of reference. The sum score comprises all items and scores of 5, 10, and 15 indicated mild, moderate, and severe levels of anxiety symptoms. In the present study, the participants who scored above or equal to ten on the GAD-7 questionnaire, they were considered as having anxiety symptoms.

Social support was assessed by using the Oslo 3-item social support scale which was used several studies. It provides a brief measure of social support and functioning and is considered to be one of the best predictors of mental health. It covered different levels of social support by measuring the number of people the respondents feel close to, the interest and concern showed by others, and the ease of obtaining practical help from others. To OSS-3, total scores were calculated by adding up the raw scores for each item. The sum score scale ranges from 3 to 14 and has three broad categories: “Poor social support”3–8, “Moderate support” 9–11, and “Strong support” 12–14 [26–28].

Substance use factors were assessed using WHO’s Alcohol, Smoking, and Substance Involvement Screening Test (ASSIST), which is developed by the World Health Organization and its Cronbach’s alpha with 0.80, sensitivity of 80%, and specificity of 71%.

Perceived threat to life was measured using Perceived Stress Scale (PSS) with its scores, which ranges from 0 to 40 with higher scores indicating higher perceived stress, low perceived stress scores range from 0–13 on PSS, moderate perceived stress scores range from 14–26 on PSS and high perceived stress scores ranging from 27–40 on PSS-10 items [29]. Hence this tool was validated and its Cronbach’s alpha value of internal consistency 0.80(*n* = 387) [30] and in this study the alpha value found to be 0.89.

An outcome variable, an acute stress disorder, was carried out by using acute stress disorder scale (ASDS). It has 19 items that comprised the ASDS include five dissociative, four avoidance, four re-experiencing, and six arousal symptom. Its score for each question varies from 1 to 5 (1 = not at all, 2 = mildly, 3 = medium, 4 = quite, 5 = very much), with a result range of 19 to 95, it was a version from acute stress disorder interview based on DSM-IV 1994. The ASDS is scored by summing the scores for all items and based on the normal cut-off previous study in South Africa, persons who scored ≥ 56 on the ASDS-19 questionnaire were considered as having probable acute stress disorder in which the person who exposed by sudden physical injury from a car accident, gunshot, burn, falling from upstairs/tree, physical fighting during three days to one month after trauma exposure. The cut-off point was greater than the medium of Likert scale was adopted and it was also the same as mean value of this study [31]. It has demonstrated an excellent internal consultancy Cronbach’s alpha of 0.96 in Texas, United States of America [31]. Moreover, ASDS has good sensitivity (90%) and specificity (83%) for determining acute stress disorder against the ASD interview on 99 civilian trauma survivors [32].

### Data analysis

The completed questionnaire was checked for completeness and then was coded, recoded, and entered into Epi-info version 7 statistical programs and then was exported to SPSS version 20 for analysis. Binary logistic regression analysis was used to identify factors associated with ASD whose *p*-values were less than 0.20 levels. All variables, which were significantly associated with binary logistic regression entered into the multivariable logistic regression. Finally, the variables that had an independent association with acute stress disorder were declared based on 95% CI and *P* < 0.05.

## Results

### Socio-demographic and clinical characteristics of study participants

A total of (*n* = 422) physically traumatized patients have completed the study with a response rate of 100%. The median age of participants was 36 (SD ± 13) with age ranging from 18 to 72 years. Out of the study participants, 289(68.5%) were males, and more than half of participants 52.6% were singles. Of the participants about three-fourths 76.3% belong to Orthodox Christian followers and 84.1% of them were Amhara by their ethnicity. More than two-fifths of (43.1%) of participants were unable to read and write, half of respondents (50.9%) were rural dwellers while 32.8% of the participants were farmers. Regarding the clinical characteristic of study subjects; over one-third (33.2%) of the study participants experienced gunshot traumatic events and 20.4% had anxiety symptoms. Of the participants, more than one-tenth 12.3% had taken psychiatric medication, 36% had past traumatic history, 7.6% had past psychiatry illness, 6.2% had the chronic medical illness, and 5.7% had family history of psychiatry illnesses (Table 1).

**Table 1:** Socio-demographic and clinical characteristics of the study participants at Gondar and Felege-Hiwot comprehensive specialized hospitals, Northwest Ethiopia (*n* = 422)

Variables	Categories	Frequency	Percent
Sex	Male	289	68.5
	Female	133	31.5
Age group	18–20 years	63	14.9
	21–29 years	99	23.5
	30-39 years	93	22.0
	40–49 years	92	21.8
	50–59 years	46	10.9
	> 59	29	6.9
Marital status	Married	134	31.8
	Single	222	52.6
	Divorced	54	12.8
	Separated/widowed	12	2.8
Religion	Orthodox	322	76.3
	Muslim	69	16.4
	Protestant/Catholic	31	7.3
Ethnicity	Amhara	355	84.1
	Oromo/Tigre	47	11.2
	Other^a^	20	4.7
Education	Unable to read and write	182	43.1
	1–8 grade	105	24.9
	9–10 grade	41	9.7
	11–12 grade	33	7.8
	Diploma	25	5.9
	Degree and above	26	8.5
Residence	Rural	215	50.9
	Urban	207	49.1
Occupation	Employee	63	14.9
	Merchant	99	23.5
	Farmer	138	32.7
	Student	49	11.6
	House wife	53	12.6
	Others^b^	19	4.5
Past psychiatry history	Yes	30	7.1
	No	394	92.9
Family history of mental illness	Yes	24	5.7
	No	398	94.3
History of psychiatry medication	Yes	52	12.3
	No	370	87.7
Chronic medical illness	Yes	26	6.2
	No	396	93.8
Anxiety	Yes	86	20.4
	No	336	79.6
Past history of trauma	Yes	36	8.5
	No	386	91.5
Types of trauma	Car accident	124	29.4
	Gun shut	140	33.2
	Falling down	64	15.2
	Burn	51	12.1
	Physical fight/ bit	43	10.2

Other^a^ = Kemant, Agew and Gumz: Other^b^ = Daily laborer, Driver

### Psychosocial and substance use characteristics of the study participants

Findings indicate that that one-third of the participants (30.6%) had low perceived stress (based on the cut-off score less than or equal to 13 as measured by perceived stress scale-10). Over one-thirds 38.9% of study participants had poor social support. Regarding alcohol use, over two-fifth 43.1% was drinking once in their lifetime while alcohol is being currently consumed by 17.8% of them during the study time. A smaller proportion of the participants (9.1%) were found to be current chewers of khat during the study period (also called chat in Ethiopia) (Table 2).

**Table 2:** Psychosocial and substance characteristics of the study participants at Gondar and Felege-Hiwot Comprehensive Specialized Hospital, Northwest, Ethiopia (*n* = 422)

Variables	Categories	Frequency	Percent
Social support	Poor support	164	38.9
	Moderate support	138	32.7
	Strong support	120	28.4
Perceived life threatens	Low	271	64.2
	Moderate	129	30.6
	High	22	5.2
Ever use			
Alcohol	Yes	182	43.1
	No	240	56.9
Khat	Yes	46	10.9
	No	376	89.1
Current use			
Alcohol	Never	251	59.7
	Once and twice	48	11.4
	Weekly	35	8.3
	Monthly	12	2.8
	Daily	75	17.8
Khat	Never	384	91.0
	Once and twice	5	1.2
	Weekly	5	1.2
	Monthly	2	0.5
	Daily	26	6.2

#### Types of trauma and acute stress disorder by sex

**Fig. 1:**
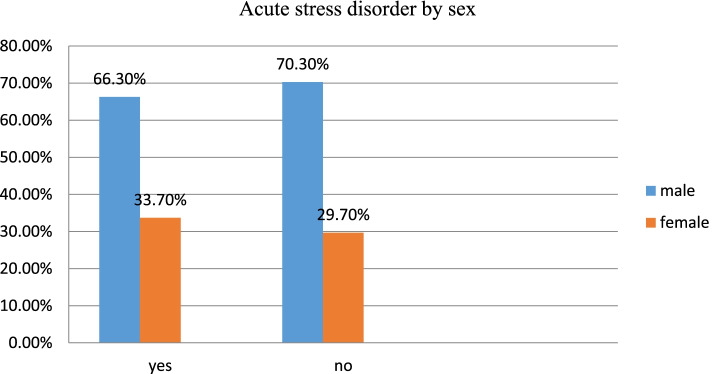
Description of types of trauma by sex among study participants at the University of Gondar and Felege-Hiwot comprehensive specialized hospitals, Northwest Ethiopia, 2020 (*n* = 422)

**Fig. 2:**
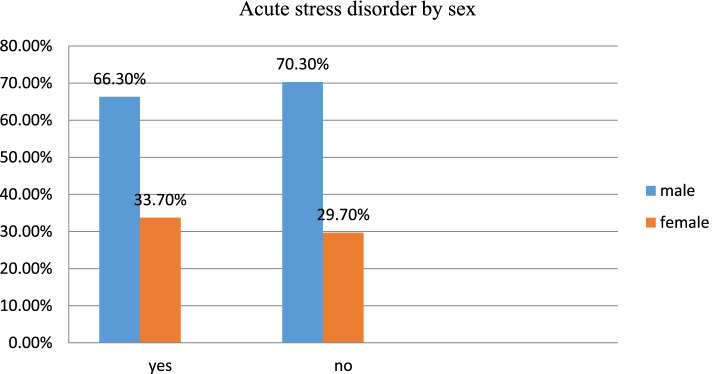
Description of acute stress disorder by sex among study participants at the University of Gondar and Felege-Hiwot comprehensive specialized Hospitals, Northwest Ethiopia, 2020 (*n* = 422)

Out of the study participants, over two-fifth (43.6%) of female participants experienced traumatic event related to car accident, while 41.9% of male experienced car accident traumatic events. Gun shot was reported to be the types of traumatic event reported by 41% of male and 18.7% of female participants exposed for traumatic events related to falling down from trees/cliffs/upstairs. Similarly, one-tenth of female experienced burn and 11.3% of male reported physical fight/bit traumatic events (Fig. 1). Of the study participants, two in every three males had acute stress disorder while one in every three females had encountered acute stress disorder (Fig. 2).

#### Prevalence and associated factors of probable acute stress disorder

In this study, the prevalence of probable acute stress disorder among study participants was found to be 45.0% (95% CI: 40.2–49.6). Specifically, the prevalence from a clinical population in the two sites Gondar Comprehensive and Felege-Hiwot Specialized hospitals were 43.8% and 47.5%, respectively. Residence, types of trauma, past history of trauma, chronic medical illnesses, and history of psychiatry medication, history of psychiatry illnesses, anxiety, social support, and perceived threat in life were factors selected further multivariable analysis because these variables have satisfied a preliminary assumptions to be came candidate variables (their association) with acute stress disorder shows (*P* < 0.2 in the bivariable logistic regression).

This enables us to see the association of each predictor variable with outcome variables while controlling the potential confounding factors. After controlling potential confounding factors in the multivariable analysis, findings showed that the previous history of trauma, past psychiatry illnesses, anxiety, poor and moderate social support, and moderate stress to life threaten events were factors significantly associated with probable acute stress disorder *p*-value less than 0.05. From clinical related factors exposure to past history of trauma has demonstrated 3.46 times more likely to develop probable acute stress disorder compared to participants who did not have an exposure of previous history of trauma (AOR = 3.46, 95% CI: 1.01–11.80), and the odds of showing probable acute stress disorder among participants with past history of mental illnesses demonstrated 3.02 times higher than those who did not past history of psychiatry illness (AOR = 3.02, 95% CI: 1.15–7.93). In the present study, those participants with anxiety symptoms demonstrated 2.38 times more likely at risk of developing probable ASD compared to participants who did not have anxiety symptoms (AOR = 2.38, 95% CI: 1.30–4.38). Participants who had poor and moderate social support were 4.07 and 4.56 times more likely to develop acute stress disorder compared to their counterparts (AOR = 4.07, 95% CI: 2.20–7.52) and (AOR = 456, 95%, CI: 2.43–8.52), respectively. The odds of showing acute stress disorder among participants’ with experienced a moderate perceived threat to life demonstrated 2.74 times higher odds of developing probable ASD compared to participants who did not have the perceived threat for life (AOR = 2.74, 95% CI: 1.64–4.60). When bivariate analysis without consideration of potential confounding was taken into account, participants who were exposed to traumatic events were associated with car accident demonstrated more than twice at higher odds of developing ASD compared to a physical fight/bit traumatized (COR = 2.06, 95% CI: 1.02–4.22). Similarly, participants who encountered falling down have shown 2.4 times more exposure to ASD compared to those who encountered a physical fight/ bit (COR = 2.40, 95% CI: 1.08–5.33) *p* < 0.05 (Table 3).

**Table 3:** Factors associated with ASD among physically traumatized patients at Gondar and Felege-Hiwot Comprehensive Specialized Hospital, Northwest, Ethiopia (*n* = 422)

Variables	ASD		COR (95%CI)	AOR (95%,CI)
	Yes	No		
Marital status				
Married	48	86	1	1
Single	103	119	1.60 (0.99, 2.41)	1.30(0.77, 2.19)
Divorce	34	20	3.04(1.60, 5.87)	2.03 (0.93, 4.43)
Separated/widowed	5	7	1.28(0.39, 4.25)	0.39 (0.07, 2.11)
Residence				
Rural	88	127	1	1
Urban	102	105	1.40 (0.95, 2.06)	0.92 (0.57, 1.50)
Types of trauma				
Car accident	65	59	2.06(1.02, 4.22)	1.97(0.830, 4.69)
Gunshot	53	87	1.13(0.56, 2.32)	1.15 (0.49, 2.71)
Falling down	36	28	2.40(1.08, 5.33)	1.78 (0.69, 4.60)
Burn	21	30	1.30(057, 3.02)	0.82 (0.30, 2.29)
Physical fight/bit	15	28	1	1
Psychiatry medication				
Yes	41	11	5.52 (2.75, 11.10)	2.40 (0.89, 6.48)
No	149	221	1	1
Chronic medical illness				
Yes	20	6	4.43(1.74, 11.27)	1.44(0.44, 4.76)
No	170	226	1	1
History of traumatic events				
Yes	31	5	8.85(3.57, 23.26)	3.46 (1.01, 11.80)*
No	159	227	1	1
History of psychiatry				
Yes	24	8	4.04 (1.77, 9.23)	3.02(1.15, 7.92)*
No	166	224	1	1
Anxiety				
No	127	209	1	1
Yes	63	23	4.508(2.66, 7.62)	2.38 (1.30, 4.38)**
Social support				
Poor support	90	74	4.62(2.70, 7.90)	4.07 (2.20, 7.51)***
Moderate support	75	63	4.52(2.60, 7.87)	4.56(2.43, 8.52)***
Strong support	25	95	1	1
Perceived threat to life				
Low stress	93	178	1	1
Moderate stress	81	48	3.23(2.09, 5.00)	2.74 (1.64, 4.60)***
High stress	16	6	5.10(1.93, 13.48)	1.873 (0.57, 6.140)

^*^p-value less than 0.05; **p-value less than 0.01; ***p-value less than 0.001

## Discussions

In the present hospital-based institutional cross-sectional study, the interesting findings were obtained. Participants having a history of exposure to traumatic events in the past, history of psychiatric illness in the past, poor and moderate social support, and exposure to moderate stress to threatening life events were significantly associated with probable ASD of participants. In this section, the major findings of the current study are discussed in light of previous empirical evidence from the literature. Attempts were made to provide sufficient justifications on unique outcomes of this study in a way it can best explain the study prevailing cultural, political as well as the socioeconomic context of Ethiopia at the current time. Thus, detail discussion of findings presented in the subsequent paragraphs.

Acute stress disorder is the most common and the main public health matter after an exposed physical trauma, and it was a serious problem for traumatized adult people who met the criteria for acute stress disorder. This study showed that the prevalence of probable acute stress disorder among study participants was found to be 45%. The magnitude of this study is in line with other studies conducted in South Africa, 40.9% [33] and the United Kingdom 40.6% [34]. However, this current study was lower than the study done in the West mead hospital 60% and New Orleans, 62% [35, 36], respectively. The possible reason for the discrepancy might be differences in study design, instruments, the nature of the accident, and sample size used. In West mead hospital, maybe due to difference in sample size for only 51 motor vehicle accident participants, an instrument that was an acute stress disorder interview which might be overestimating the prevalence of cases by interpretation of the scores, in study design that was a prospective study [35]. The discrepancy in New Orleans study may be due to difference in sampling method (convenience sampling), study setting that was conducted in the community-based survey may increase prevalence cases by unable get early treatment and lack of availability of special care with health professions and nature of trauma that was a natural disaster (flooding) [36]. Moreover, the current unstable crisis in Ethiopia, such as ethnic clash and fragile protection of human rights and may add stress besides personal life events that victims are encouraging in their daily life may be contribute for high prevalence of probable ASD in this study.

On the contrary, this finding of the current study is higher than those of other studies conducted in London 19%, Australia 28%, in emergency Clinics University of Manchester 21%, Japan 16%, and 23.6% among Johns Hopkins Burn Center [13, 37–40]. The variations seen in the above magnitude of ASD reported in different countries compared to the prevalence of this disorder in the current study might be due to differences in methods and procedure of data collection followed, study setting, types of study design, application of measurement tools which was not properly adapted to suit the cultural context of some studies differences in the types of population and the prevailing socio-cultural and economic contrasts between Ethiopia and the other countries. More importantly, one possible justification for the relatively higher prevalence of acute stress disorder is that these two hospitals where cases that were highly affected by severing forms of traumatic events from the northwest region of Ethiopia were referred to these settings. In addition to the intact economic poverty and inadequacy of treatment, the chance of those who could develop acute stress disorder is higher in Ethiopia at the present, because the country in general and the surrounding society in Gondar and Bahir Dar, in particular, are recently exposed for multifaceted problems of human rights violations, such as domestic abuse, aggression, looting and consequently prone to physical and psychological trauma. The question remains despite the availability of culturally intact protective psychosocial factors such as rich social support network, religious source of coping, adherence to age-old and civilized cultural norm that had existed in Ethiopia and currently in place, what lead Ethiopians develop higher on ASD warrants further investigation.

Among significantly associated factors with acute stress disorder; history of participants who were exposed to traumatic events in the past were about over three times more likely to develop acute stress disorder compared to those participants who had no the history of past trauma. The current finding is in line with the theory, which explains the development of acute stress disorder for trauma survivors just following their exposure to different forms of life-shattering events, which are traumatic by their nature shortly after they have experienced it [2]. The present finding is also supported by studies conducted in South Africa and Australia [34, 41]. Another possible reason might be due to unresolved traumatic events in the past might be affected their thoughts or emotions response to something that reminds in the past traumatic situations which is more susceptible to acute stress disorder while exposed such traumatic events again.

The respondents with having history of psychiatry illness were three times greater likelihood to develop acute stress disorder compared to the participants who had no the history of psychiatry illness. This finding is supported by studies conducted in South Africa and Denmark [2, 34]. One possible justification is that patients with past psychiatric illness could be exposed to neuro- chemical imbalance and neuronal dysfunction compared to people who had no past psychiatry illness [42]. Hence, when people having neuron-chemical imbalance because of previous psychiatric illness encountered traumatic events, these accelerate the onset of acute stress disorder [8]. Alternatively, people having an experience of psychiatric illness in the past were having more chances to develop acute stress disorder, because they were classically conditioned and hence even a simple traumatic event can trigger ASD [8]. Moreover, absence of appropriate psychiatric medication, absence of culturally relevant psychosocial intervention, a total absence of psychotherapy, poor adherence to medication the associated with stigma due to the prior illness, and the consequent poor social interaction for fear of discrimination, may worsen survivors of trauma with past psychiatric illness to be susceptible to acute stress disorder.

Participants who had anxiety were two times odds to develop acute stress disorder compared to respondents who had not anxiety. This is supported by study conducted at South Africa and West mead hospital [34, 35]. The possible justification for the findings in the current study can be explained by the fact that having an additional psychiatric diagnosis (i.e. co-morbidly of additional psychiatric disorder) may increase likelihood of developing acute stress disorder and poor durable health outcomes including impaired functioning and lower self-esteem as well as poor quality of life, as evidenced in previous pieces of evidence [11, 12]. Unresolved anxiety, of participants acquired before their admission to hospitals, may contribute to the odds the development of acute stress disorder.

The present study demonstrated that participants who had poor and moderate social support were more than four times more possibility to develop acute stress disorder compared to the participants who have strong social support. This finding is consistent to establish and consistent previous finding that indicated the association between received social support and vulnerability to ASD during intermediate period of the trauma, but perceived availability of social support was not related to distress immediately following trauma nor five months afterward, such as findings reported in Denmark [43]. Besides, individuals who received inadequate and poor social support may not likely develop effective coping mechanisms to the adverse effects of trauma, and hence such people are susceptible to ASD.

The odds of developing acute stress disorder of the participants among those with moderate perceived stress to life-threatening events in the current study were found more than two times higher compared to the participants who had low perceived stress such an event. The present finding is supported by previous study conducted in San Diego in the United States [44]. Such findings may be justified by the fact that negative beliefs about the consequences of an adverse event are threatening to one’s life may the onset of ASD, because of irrational thinking and distorted intact belief, as consistently reported by adherents of rational emotive behavior therapists regarding the etiology of a disorder [45].

### Strength and limitations of the study

The fact that this study has come up with some vital findings regarding the magnitude and associated factors of acute stress disorder despite the fact that this topic was not studied in Ethiopia with such backdrop of the paucity of evidence about this disorder among people in Africa, the present study can be taken as the strength of the study. Making a transition and back translation of measures used in the present study can also be taken as the strength. The most important limitation of this study is the acute stress disorder scale, which was employed in the present study, was not validated in the Ethiopian context although it is widely used as a screening tool to measure ASD in other cultural contexts with minor modifications and adaptation. Besides, since the present study was based on a cross-sectional study making an inference of causal relationship between independent and outcome variables (i.e. acute stress disorder) is not possible.

## Conclusions

Findings of this study indicated that the prevalence of acute stress disorder among participant patients exposed to multiple forms of traumatic events was considerably high. History of trauma and past psychiatric illness, depression, poor and moderate social support, and moderate perceived stress were factors significantly associated with acute stress disorder. The ministry of health and other concerned health organizations may find the current finding useful for early detection, prevention, and intervention strategies to minimize the factor of acute stress disorder in trauma survivors. Researchers should conduct a further study on acute stress disorder and its multiple impact on physical, mental, occupational and social functioning of people exposed for varieties of traumatic events using a different approach, including other study variables and designs, such as prospective cohort and case control in this study area as well as different parts of the country for further exploration of the problem.

## Data Availability

The datasets generated during and analyzed the current study are not publicly available but are available from the corresponding author on reasonable request.

## Abbreviations

AOR: Adjusted Odds Ratio
ASD: Acute Stress Disorder
ASDS: Acute Stress Disorder Scale
CI: Confidence Interval
DSM: Diagnostic and Statistical Manual for Mental Disorder
EPI-Info: Epidemiological Information
GAD-7: Generalized Anxiety Disorder 7-item (GAD-7) scale
OSS-3: Oslo 3 Items Social Support Scale
UOGCSH: University Of Gondar Comprehensive Specialized Hospital
RTA: Road Traffic Accident
SPSS: Statistical Package for Social Sciences
UOG: University of Gondar
WHO: World Health Organization

## References

[1] Rapsang AG, Shyam DC (2015). Scoring systems of severity in patients with multiple trauma. Cirugía Española (English Edition).

[2] Karakaya I, Çolak B (2007). Çocuk ve ergenlerde travma sonrası stres bozukluğu ve adli tıbbi değerlendirme. Adli Tıp Bülteni.

[3] Peden M, Scurfield R, Sleet D, Mathers C, Jarawan E, Hyder A (2004). World report on road traffic injury prevention: World Health Organization.

[4] Bashah DT, Dachew BA, Tiruneh BT (2015). Prevalence of injury and associated factors among patients visiting the Emergency Departments of Amhara Regional State Referral Hospitals, Ethiopia: a cross-sectional study. BMC Emerg Med.

[5] Moshiro C, Heuch I, Åstrøm AN, Setel P, Hemed Y, Kvåle G (2005). Injury morbidity in an urban and a rural area in Tanzania: an epidemiological survey. BMC Public Health.

[6] Tayeb SE, Abdalla S, Mørkve O, Heuch I, Van den Bergh G (2014). Injuries in Khartoum state, the Sudan: a household survey of incidence and risk factors. Int J Inj Contr Saf Promot.

[7] Tsegaye F, Abdella K, Ahmed E, Tadesse T, Bartolomeos K (2010). Pattern of fatal injuries in Addis Ababa, Ethiopia: a one-year audit. East and Central African J Surg.

[8] Edition F (2013). Diagnostic and statistical manual of mental disorders. Am Psychiatric Assoc.

[9] Bryant RA (2010). Acute stress disorder as a predictor of posttraumatic stress disorder: a systematic review. J Clin Psychiatry.

[10] Ginzburg K, Ein-Dor T (2011). Posttraumatic stress syndromes and health-related quality of life following myocardial infarction: 8-year follow-up. Gen Hosp Psychiatry.

[11] von Känel R, Hari R, Schmid J-P, Saner H, Begré S (2011). Distress related to myocardial infarction and cardiovascular outcome: a retrospective observational study. BMC Psychiatry.

[12] Bryant RA, Harvey AG (1999). The influence of traumatic brain injury on acute stress disorder and post-traumatic stress disorder following motor vehicle accidents. Brain Inj.

[13] Brewin CR, Andrews B, Rose S, Kirk M (1999). Acute stress disorder and posttraumatic stress disorder in victims of violent crime. Am J Psychiatry.

[14] Association AP (2013). Diagnostic and statistical manual of mental disorders. BMC Med.

[15] Thombs BD, Fauerbach JA, McCann UD (2005). Stress disorders following traumatic injury: assessment and treatment considerations. Primary Psychiatry.

[16] Bryant R. Acute stress disorder in adults: Epidemiology, pathogenesis, clinical manifestations, course, and diagnosis. 2019.

[17] Bryant RA (2011). Acute stress disorder as a predictor of posttraumatic stress disorder: a systematic review. J Clin Psychiatr.

[18] Frommberger UH, Stieglitz R-D, Nyberg E, Schlickewei W, Kuner E, Berger M (1998). Prediction of posttraumatic stress disorder by immediate reactions to trauma: a prospective study in road traffic accident victims. Eur Arch Psychiatry Clin Neurosci.

[19] Tsay SL, Halstead MT, McCrone S (2001). Predictors of coping efficacy, negative moods and post-traumatic stress syndrome following major trauma. Int J Nurs Pract.

[20] Barton KA, Blanchard EB, Hickling EJ (1996). Antecedents and consequences of acute stress disorder among motor vehicle accident victims. Behav Res Ther.

[21] Flach F. Acute stress disorder: A handbook of theory, assessment, and treatment. Am J Psychiat. 2000;157(10):1713.

[22] Meister RE, Weber T, Princip M, Schnyder U, Barth J, Znoj H (2015). Resilience as a correlate of acute stress disorder symptoms in patients with acute myocardial infarction. Open heart.

[23] Alemu WG, Malefiya YD (2014). Prevalence and associated factors of common mental disorders among patients admitted in Gondar university hospital medical and surgical wards, northwest Ethiopia. J Neurosci.

[24] Belayihun B, Mavhandu-Mudzusi AH (2019). Effects of surgical repair of obstetric fistula on severity of depression and anxiety in Ethiopia. BMC Psychiatry.

[25] Gelaye B, Williams MA, Lemma S, Deyessa N, Bahretibeb Y, Shibre T (2013). Validity of the patient health questionnaire-9 for depression screening and diagnosis in East Africa. Psychiatry Res.

[26] Abiola T, Udofia O, Zakari M (2013). Psychometric properties of the 3-item oslo social support scale among clinical students of Bayero University Kano. Nigeria Malaysian J Psychiatr.

[27] Emebet G. The prevalence of Psychological Distress and associated factors among Caregivers of Schizophrenia Outpatients: The Case of Amanuel Mental Specialized Hospital. 2015. http://etd.aau.edu.et/handle/123456789/11050.

[28] Getnet B, Medhin G, Alem A (2019). Symptoms of post-traumatic stress disorder and depression among Eritrean refugees in Ethiopia: identifying direct, meditating and moderating predictors from path analysis. BMJ Open.

[29] Cohen S, Kamarck T, Mermelstein R. Perceived stress scale. Measuring stress: A guide for health and social scientists. 1994;10(2):1–2.

[30] Manzar MD, Salahuddin M, Peter S, Alghadir A, Anwer S, Bahammam AS (2019). Psychometric properties of the perceived stress scale in Ethiopian university students. BMC Public Health.

[31] Edmondson D, Mills MA, Park CL (2010). Factor structure of the acute stress disorder scale in a sample of Hurricane Katrina evacuees. Psychol Assess.

[32] Bryant RA, Moulds ML, Guthrie RM (2000). Acute Stress Disorder Scale: a self-report measure of acute stress disorder. Psychol Assess.

[33] Ophuis RH, Olij BF, Polinder S, Haagsma JA (2018). Prevalence of post-traumatic stress disorder, acute stress disorder and depression following violence related injury treated at the emergency department: a systematic review. BMC Psychiatry.

[34] Bryant RA, Panasetis P (2001). Panic symptoms during trauma and acute stress disorder. Behav Res Ther.

[35] Mills MA, Edmondson D, Park CL (2007). Trauma and stress response among Hurricane Katrina evacuees. Am J Public Health.

[36] Bryant RA, Friedman MJ, Spiegel D, Ursano R, Strain J (2011). A review of acute stress disorder in DSM-5. Focus.

[37] Holeva V, Tarrier N, Wells A (2001). Prevalence and predictors of acute stress disorder and PTSD following road traffic accidents: Thought control strategies and social support. Behav Ther.

[38] Harvey AG, Bryant RA (2002). Acute stress disorder: a synthesis and critique. Psychol Bull.

[39] Roberge M-A, Dupuis G, Marchand A (2008). Acute stress disorder after myocardial infarction: prevalence and associated factors. Psychosom Med.

[40] Harvey AG, Bryant RA (1999). Predictors of acute stress following motor vehicle accidents. J Trauma Stress.

[41] Elklit A, Brink O (2004). Acute stress disorder as a predictor of post-traumatic stress disorder in physical assault victims. J Interpers Violence.

[42] Holbrook TL, Hoyt DB, Coimbra R, Potenza B, Sise M, Anderson JP (2005). High rates of acute stress disorder impact quality-of-life outcomes in injured adolescents: mechanism and gender predict acute stress disorder risk. J Trauma Acute Care Surg.

[43] Cook JD, Bickman L (1990). Social support and psychological symptomatology following a natural disaster. J Trauma Stress.

[44] Kangas M, Henry JL, Bryant RA (2007). Correlates of acute stress disorder in cancer patients. J Trauma Stress.

[45] Ellis A, Dryden W (2007). The practice of rational emotive behavior therapy: Springer publishing company.

